# Modeling and simulations to support dose selection for eslicarbazepine acetate therapy in pediatric patients with partial-onset seizures

**DOI:** 10.1007/s10928-018-9596-7

**Published:** 2018-06-09

**Authors:** Soujanya Sunkaraneni, Elizabeth Ludwig, Jill Fiedler-Kelly, Seth Hopkins, Gerald Galluppi, David Blum

**Affiliations:** 1 Sunovion Pharmaceuticals Inc., 84 Waterford Drive, Marlborough, MA 01752 USA; 2 Cognigen Corporation, a Simulations Plus Company, 1780 Wehrle Drive #110, Buffalo, NY 14221 USA

**Keywords:** Epilepsy, Pediatric, Modeling and simulations, Population pharmacokinetic model, Eslicarbazepine

## Abstract

Modeling and simulations were used to support body weight-based dose selection for eslicarbazepine acetate (ESL) in pediatric subjects aged 4–17 years with partial-onset seizures. A one-compartment pediatric population pharmacokinetic model with formulation-specific first-order absorption, first-order elimination, and weight-based allometric scaling of clearance and distribution volume was developed with PK data from subjects 2–18 years of age treated with ESL 5–30 mg/kg/day. Covariate analysis was performed to quantify the effects of key demographic and clinical covariates (including body weight and concomitant use of carbamazepine, levetiracetam, and phenobarbital-like antiepileptic drugs [AEDs]) on variability in PK parameters. Model evaluation performed using a simulation-based visual predictive check and a non-parametric bootstrap procedure indicated no substantial bias in the overall model and in the accuracy of estimates. The model estimated that concomitant use of carbamazepine or phenobarbital-like AEDs with ESL would decrease the exposure of eslicarbazepine, and that concomitant use of levetiracetam with ESL would increase the exposure of eslicarbazepine, although the small effect of levetiracetam may not represent a true difference. Model-based simulations were subsequently performed to apply target exposure matching of selected ESL doses for pediatric subjects (aged 4–17 years) to attain eslicarbazepine exposures associated with effective and well-tolerated ESL doses in adults. Overall, model-based exposure matching allowed for extrapolation of efficacy to support pediatric dose selection as part of the submission to obtain FDA approval for ESL (adjunctive therapy and monotherapy) in subjects aged 4–17 years, without requiring an additional clinical study.

## Introduction

Eslicarbazepine acetate (ESL) is a once-daily (QD), oral antiepileptic drug (AED) for the treatment of partial-onset seizures (POS) in patients ≥ 4 years of age in the USA, and in patients > 6 years of age in Europe. Following oral administration, ESL undergoes rapid first-pass hydrolysis to the primary active metabolite eslicarbazepine and its glucuronide metabolites, which together account for 94% of oral systemic exposure; R-licarbazepine and oxcarbazepine are minor active metabolites [1].

A recent analysis conducted by the US Food and Drug Administration (FDA), the University of Maryland, and the Pediatric Epilepsy Academic Consortium for Extrapolation (PEACE), provided evidence across AEDs that exposure–response relationships are preserved between adult and pediatric subjects (aged ≥ 4 years) with POS [2]. From this analysis, the FDA set three criteria for acceptable extrapolation of the effectiveness of AEDs in adult subjects to pediatric subjects (≥ 4 years) with POS: (1) an approved indication for treatment of POS in adults; (2) a pharmacokinetic (PK) analysis that allows selection of dosing regimens for pediatric patients aged 4–17 years resulting in exposures similar to those that have been demonstrated to be safe and effective in adults; and (3) a long-term, open-label safety study in pediatric patients ≥ 4 years [3–8]. Since ESL is approved for the treatment of POS in adults, the first FDA requirement is met. Also, placebo-controlled studies up to 12 weeks in duration, with long-term, open-label extensions, have demonstrated that ESL is well-tolerated in pediatric patients ≥ 4 years of age [9–11], thus satisfying the third FDA requirement. Therefore, this paper focuses on the PK modeling and simulation strategies implemented to satisfy the second FDA requirement. Previously developed population PK (PPK) and exposure–response models describing the PPK, safety, and efficacy of ESL (adjunctive therapy and monotherapy) in adults with POS [12, 13] were utilized for this purpose. In addition, PK data from two multiple-dose studies of adjunctive ESL in pediatric subjects aged ≥ 2 years with POS (Studies BIA-2093-202, BIA-2093-305) supported pediatric PPK model development, which allowed subsequent targeted exposure matching.

The strategy applied in this analysis was consistent with FDA recommendations for bridging efficacy data from adult to pediatric populations, as described in the pediatric study decision tree in the FDA guidance document, as follows: when there is an assumption of similar disease progression and similar response to intervention, and it is reasonable to assume similar concentration–response in pediatric and adult subjects, it is appropriate to conduct PK studies to select doses that result in exposures similar to those in adults [6].

The Pediatric Research Equity Act (PREA) requires that all applications to the FDA for a new active ingredient contain a pediatric assessment [14]. As such, the safety and effectiveness of ESL for POS must be demonstrated in all relevant pediatric subpopulations with data to support dosing and administration in these patients.

**Fig. 1:**
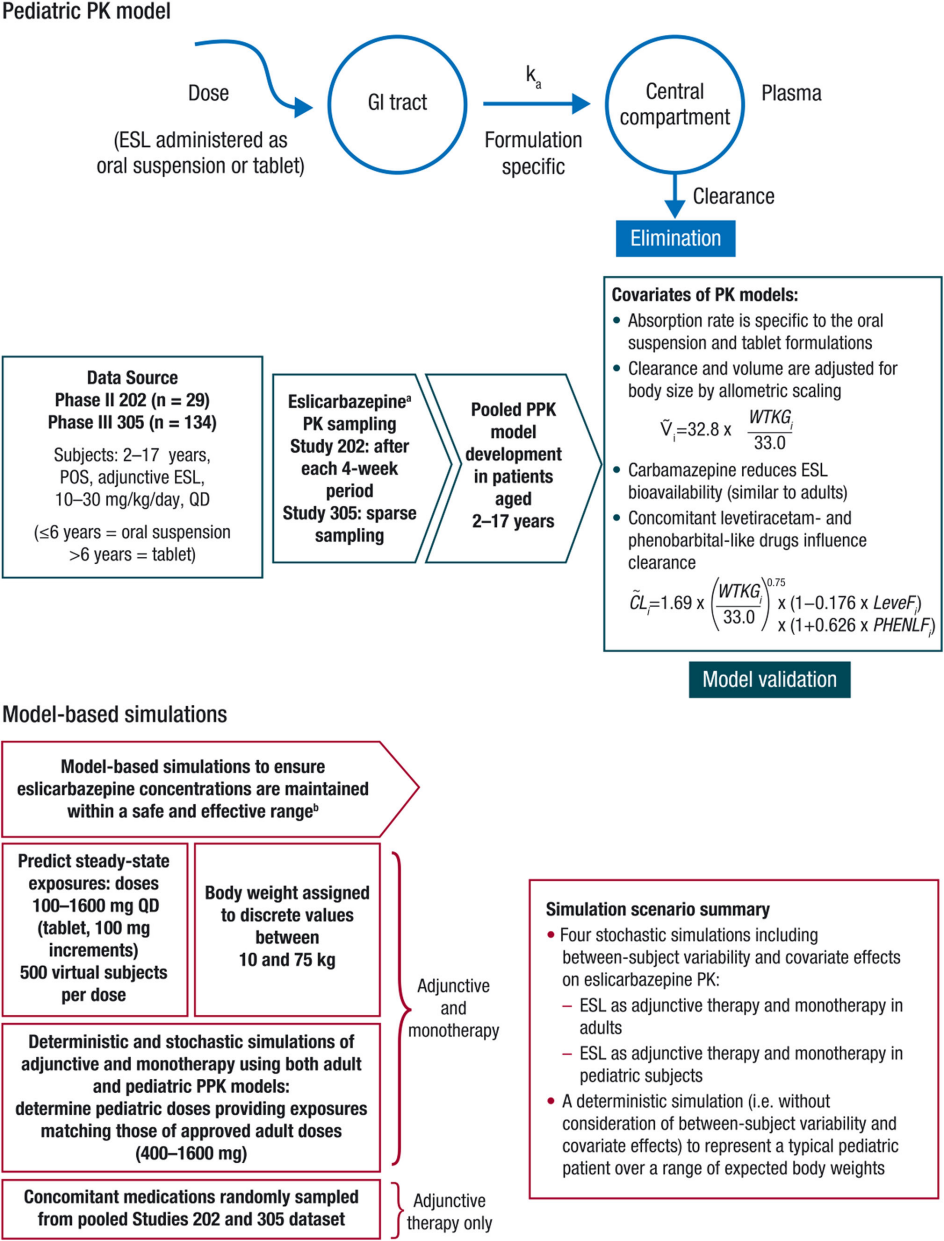
Modeling and simulation strategies applied to support ESL dose selection for pediatric subjects aged 4–17 years with POS. ^a^Primary active metabolite. ^b^Based on mean minimum observed plasma concentration at steady state (C_min,ss_) predicted for 400, 800, 1200 and 1600 mg QD ESL in adults (adjunctive and monotherapy). CL, apparent oral clearance; C_min,ss_, minimum plasma concentration at steady state; ESL, eslicarbazepine acetate; GI, gastrointestinal; *i*, ith subject; k_a_, absorption rate constant; LeveF, yes/no (subject co-administered levetiracetam); PHENLF, yes/no (subject co-administered phenobarbital-like drug); PK, pharmacokinetic; POS, partial-onset seizures; PPK, population PK; QD, once daily; V, apparent volume of distribution; WTKG, weight in kg

This paper describes the PPK modeling and simulation strategy (as shown in Fig. 1) that was applied to support ESL dose selection for pediatric subjects aged 4–17 years.

## Methods

### Study data

The data used to develop the PPK model in pediatric subjects were obtained from Studies BIA-2093-202 and BIA-2093-305, hereafter referred to as Studies 202 and 305, respectively. Data from Study BIA-2093-208 were included in the safety analyses. Details of these studies are summarized in Table 1.

**Table 1:** Study data used to develop the PPK model, and to evaluate the safety of ESL in pediatric subjects

	BIA-2093-202	BIA-2093-208	BIA-2093-305
Included in model/analysis	PPK	Safety	PPK
Subjects	n = 30 Pediatric subjects aged 2–17 years	n = 120 Pediatric subjects aged 4–17 years	n = 118 Pediatric subjects aged 2–18 years
Study design	3 consecutive 4-week treatment periods	Titration: 4 weeks Maintenance: 8 weeks	Titration: 6 weeks Maintenance: 12 weeks
ESL dosage	Oral, QD 5, 15, and 30 mg/kg/day (maximum dose: 1800 mg/day) during the 1st, 2nd and 3rd 4-week treatment periods, respectively	Oral, QD Titration: 10, 20 mg/kg/day Maintenance: 30 mg/kg/day (target dose)	Oral, QD Titration: 10, 20 mg/kg/day Maintenance: 10, 20 or 30 mg/kg/day (maximum dose: 1200 mg/day)
Type of therapy	Adjunctive therapy	Adjunctive therapy	Adjunctive therapy
Plasma sample collection	~ 21 samples/subject: Frequent sampling intervals from 0.5 to 24 h post-dose on the last day of each 4-week treatment period		~ 3 samples/subject: Sparse sampling in weeks 2, 6 and 18, mostly between 12 and 24 h post dose

*ESL* eslicarbazepine acetate, *PPK* population pharmacokinetic, *QD* once daily

### Dose selection in subjects aged 4–17 years using extrapolation

#### Theoretical: development of a PPK model for pediatric subjects aged ≥ 2 years

A pediatric PPK model for subjects aged ≥ 2 years was developed using NONMEM, version 7, level 1.2 (2010; ICON Development Solutions, Ellicott City, MD) and KIWI, version 1.1 (2012; Cognigen Corporation, Buffalo, NY). Key steps of the PPK model development were: exploratory analysis of the data collected in Studies 202 and 305, application and refinement of the one-compartment model previously developed for adjunctive ESL therapy in adults [15], evaluation of covariate effects, final model refinement, and model evaluation.

The evaluation of covariates on clearance and distribution volume included the following demographic and clinical covariates: age, estimated glomerular filtration rate [16], height, race, and sex. The following concomitant AEDs (used by > 10% of the analysis population) were evaluated: carbamazepine, valproic acid, lamotrigine, topiramate, levetiracetam, phenobarbital-like inducers (phenobarbital, primidone and phenytoin). A univariate stepwise forward selection–backward elimination method was used in the covariate analysis to identify statistically significant predictors of PK variability.

The final model was evaluated using a simulation-based visual predictive check (VPC) methodology to assess concordance between the observed data and the model-based, simulated data [17]. In addition, a non-parametric bootstrap procedure was performed using the analysis dataset and the final model, to assess the accuracy of the final parameter estimates.

#### Determination of recommended dosing for pediatric subjects aged 4–17 years

Model-based simulations were performed to support dose recommendations for pediatric subjects aged 4–17 years. Previous exposure–response analyses demonstrated that minimum steady-state concentrations (C_min,ss_) best describe the relationship between eslicarbazepine exposure and efficacy in adults [18]. Furthermore, eslicarbazepine maximum concentration (C_max_) was not found to be a statistically significant predictor of the time to first occurrence of the most common adverse events in adults [19]. Therefore, C_min,ss_ was selected as the metric for target exposure matching for efficacy and was calculated using a closed-form equation in all simulations [20]. A target range for C_min,ss_ was set, based on the mean C_min_ achieved for approved adult titration (400 mg) and maintenance (800–1600 mg) doses in subjects taking ESL QD as a monotherapy. C_max_ at the highest approved ESL maintenance dose (1600 mg) was considered when setting the upper limit of the targeted exposure range. C_min,ss_ values were compared between pediatric and adult subjects and pediatric doses were selected to ensure that C_min,ss_ values would be maintained within the effective and well-tolerated range.

Stochastic simulations were performed for the four scenarios of ESL therapy (oral tablets as adjunctive therapy and monotherapy in adults and pediatric subjects) using either the adult or pediatric PPK model. A deterministic simulation was also performed to predict exposures in a typical pediatric subject over a range of expected body weights.

Virtual subjects were created by random resampling of characteristics of the actual subjects in the specific analysis. Covariate values were resampled as a vector to ensure that the distributions and covariances between covariates were realistic and consistent with the distributions observed in the analysis population. In the deterministic simulations, body weight was randomly assigned from 22 values (ranging from 10 to 74 kg, the possible weight range for pediatric subjects aged ≥ 2 years). The 22 values represented 10% increments, starting at the 3rd percentile of weight in 2-year-olds (10 kg), up to the typical weight of an adult (74 kg) [21].

The recommended dosing for pediatric subjects was determined for four categories of body weight over the range expected for subjects aged 4–17 years: 11–21, 22–31, 32–38, and > 38 kg.

## Results

### Dose selection in subjects aged 4–17 years using extrapolation

#### Development of a PPK model for pediatric subjects aged ≥ 2 years

A PPK model was developed for 146 pediatric subjects aged ≥ 2 years, using subject data (857 eslicarbazepine concentrations) from Studies 202 and 305. Demographic information is shown in Table 2.

**Table 2:** Summary of demographic and baseline characteristics for pediatric subjects with POS included in the PPK model for eslicarbazepine

Subject characteristic	Study		Overall
	BIA-2093-202	BIA-2093-305	
Mean age (years) (SD)	8.0 (4.0)	10.0 (4.0)	10.0 (4.0)
Mean height (cm) (SD)	129.0 (23.0)	136.0 (23.0)	135.0 (23.0)
Mean weight (kg) (SD)	28.9 (13.7)	36.0 (16.2)	34.7 (16.0)
Age group (years), n (%)			
2–6	11 (42.3)	27 (22.5)	38 (26.0)
7–11	8 (30.8)	45 (37.5)	53 (36.3)
12–18	7 (26.9)	48 (40.0)	55 (37.7)
Weight category (kg), n (%)			
10–19	9 (34.6)	30 (25.0)	39 (26.7)
> 19– ≤ 32	9 (34.6)	27 (22.5)	36 (24.7)
> 32– ≤ 45	5 (19.2)	31 (25.8)	36 (24.7)
> 45– ≤ 79	3 (11.5)	32 (26.7)	35 (24.0)
Race, n (%)			
White	26 (100.0)	110 (91.7)	136 (93.2)
Asian	0	10 (8.3)	10 (6.8)
Sex, n (%)			
Male	10 (38.5)	58 (48.3)	68 (46.6)
Female	16 (61.5)	62 (51.7)	78 (53.4)

*PK* pharmacokinetic, *POS* partial-onset seizures, *SD* standard deviation

The final model was a one-compartment model with formulation-specific first-order absorption, first-order elimination, and weight-based allometric scaling of clearance (CL) and volume (V). The available data supported the estimation of inter-individual variability in CL, V, and the formulation-specific absorption rate constants using exponential error models, with the variance assumed to be the same for the oral suspension and tablet formulations (Table 3). The residual variability (RV) was modeled using an additive plus proportional error model. The covariate effects included in the final PPK model were body weight (on CL and V) and concomitant administration of carbamazepine (on bioavailability), levetiracetam (on CL), and phenobarbital-like AEDs (i.e., phenobarbital, phenytoin, and primidone; on CL). The typical value of CL and V in a subject of weight *WTKG*_*i*_ can be calculated as follows:

$$ \tilde{C}L_{i} = 1.69 \times \left( {\frac{{WTKG_{i} }}{33.0}} \right)^{0.75} \times (1 - 0.176 \times LEVEF_{i} ) \times (1 + 0.626 \times PHENLF_{i} ) $$

$$ \tilde{V}_{i} = 32.8 \times \frac{{WTKG_{i} }}{33.0} $$

where *LEVEF*_*i*_ and *PHENLF*_*i*_ indicate the use (1) or not (0) of levetiracetam and phenobarbital-like AEDs, respectively.

**Table 3:** Parameter estimates, standard errors, and bootstrap-based CIs from the final PPK model for eslicarbazepine in pediatric subjects with POS

Parameter	Final parameter estimate		Interindividual variability/residual variability^a^		Bootstrap estimate^b^
	Typical value	%SEM	Magnitude	%SEM	90% CI
CL: apparent elimination clearance (L/h)	1.69	2.92	25.0 %CV	15.5	1.61, 1.77
CL: proportional shift for levetiracetam use (–)	− 0.176	25.6			− 0.247, − 0.101
CL: proportional shift for use of phenobarbital-like AEDs (–)	0.626	18.8			0.439, 0.86
V: apparent volume of distribution (L)	32.8	4.78	13.2 %CV	65.1	30.8, 45.8
KAT: first-order absorption rate constant for tablet (1/h)	0.895	Fixed	83.8 %CV	Fixed	Fixed
KAO: first-order absorption rate constant for oral suspension (1/h)	4.18	Fixed			Fixed
F1: relative bioavailability during carbamazepine use (–)	0.679	6.76	NE	NE	0.61, 0.761
RV CCV component	0.0543	11.6	328–23.3 %CV F [100–50,000]	NA	0.0443, 0.0643
RV additive component	107,000	53.2			32,300, 224,000

Minimum value of the objective function = 14,283.727

*%CV* coefficient of variation expressed as a percentage, *%SEM* standard error of the mean expressed as a percentage, *AED* antiepileptic drug, *CI* confidence interval, *CCV* constant coefficient of variation, *NA* not applicable, *NE* not estimated, *POS* partial-onset seizures, *PPK* population PK, *RV* residual variability

^a^The residual variability (%CV) was calculated using the following equation: (sqrt (F^2^ × 0.0543 + 107,000)/F) × 100, where F is the model-predicted concentration

^b^Statistics of bootstrap estimates excluded the runs that completed with error messages about early termination, rounding errors, or estimates near boundary

**Fig. 2:**
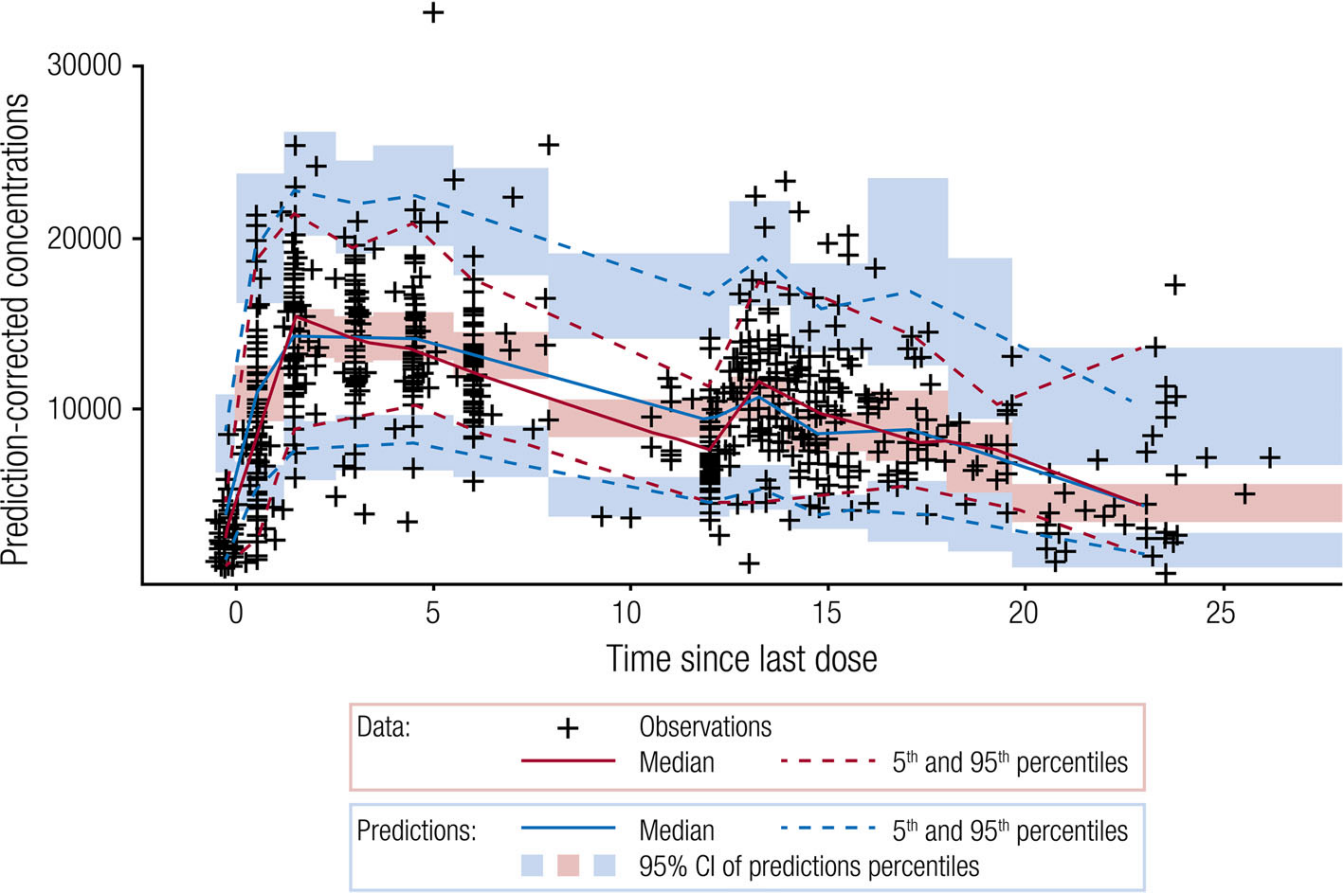
Visual predictive check of the final PPK model for ESL in pediatric subjects aged ≥ 2 years with POS. *ESL* eslicarbazepine acetate, *PPK* population pharmacokinetic, *POS* partial-onset seizures. Medians and percentiles are plotted at the median time since last dose of the data observed within each time since last dose interval

The parameter estimates (including bootstrap-based confidence intervals) for the final PPK model (Table 3) and VPC plots (Fig. 2) illustrated the good performance of this model.

Assuming administration of the same mg/kg ESL dose, a 10 and 79 kg subject were predicted to have a 25.8% lower and 24.4% higher area under the concentration versus time curve at steady-state (AUC_0–24,ss_), respectively, compared with a 33 kg subject. Eslicarbazepine CL would be 17.6% lower in pediatric subjects receiving concomitant levetiracetam compared with those not receiving this AED, which would result in a 21.4% higher AUC_0–24,ss_. Eslicarbazepine CL would be 62.6% higher in pediatric subjects receiving concomitant phenobarbital-like AEDs compared with those not receiving these AEDs, which would result in a 38.5% lower AUC_0–24,ss_. The concomitant administration of carbamazepine with ESL would decrease eslicarbazepine bioavailability and AUC_0–24,ss_ by 32.1%, compared with no concomitant carbamazepine use.

**Fig. 3:**
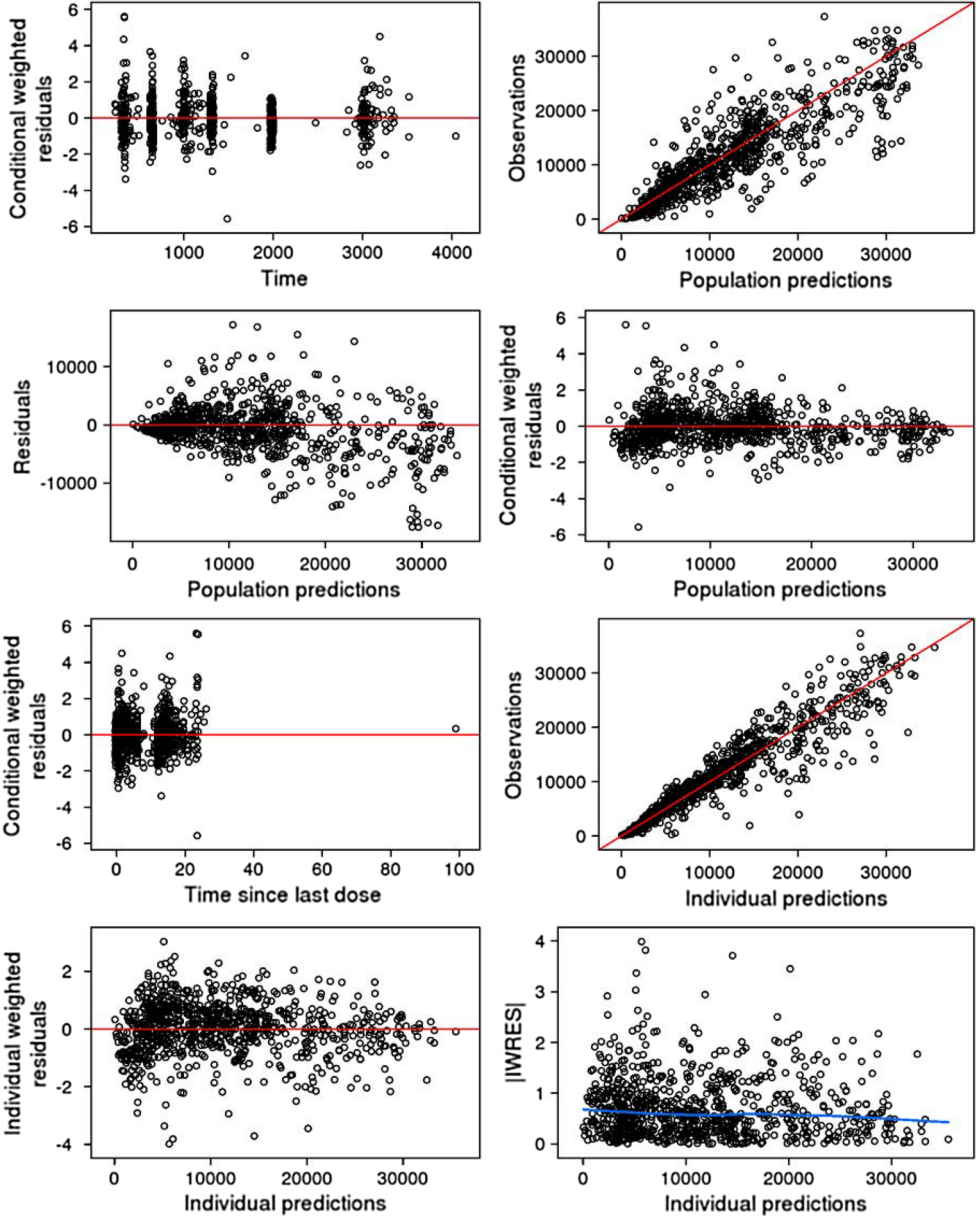
Goodness-of-fit plots for the final PPK model in pediatric subjects with POS. *PPK* population pharmacokinetic, *POS* partial-onset seizures

The final PK model described the observed data well (Fig. 3) and was used in the following simulations.

#### Determination of recommended dosing for pediatric subjects aged 4–17 years

Simulations initially determined the eslicarbazepine exposures (C_min,ss_) in adults that corresponded with effective and well-tolerated doses of ESL taken as adjunctive therapy and monotherapy during titration (400 mg QD) and maintenance (800–1600 mg QD) treatment. Mean eslicarbazepine C_min,ss_ for ESL 400 mg QD (3.7 µg/mL) was assigned as the target exposure during the titration period, and mean C_min,ss_ for ESL 800, 1200 and 1600 mg (7.4, 11 and 14.7 µg/mL, respectively) were assigned for the maintenance period.

**Fig. 4:**
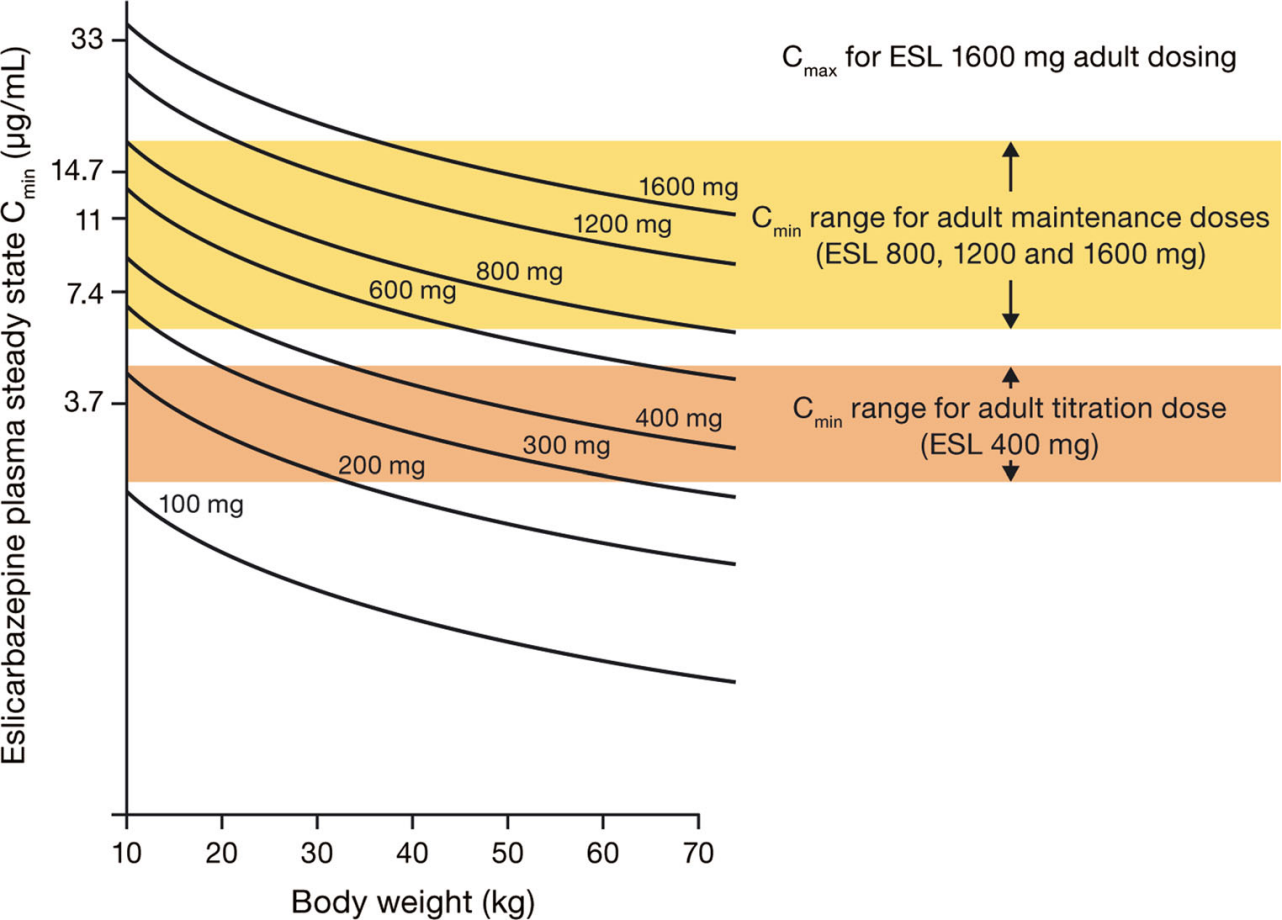
Comparison of simulated plasma concentrations of eslicarbazepine at selected dose levels (100–1600 mg/day ESL, solid lines) in pediatric subjects, relative to targeted concentration ranges for titration and maintenance (shaded regions; derived from adult exposure levels at effective and well-tolerated doses). C_min_, minimum concentration; C_max_, maximum concentration; ESL, eslicarbazepine acetate

Additional simulations predicted steady-state eslicarbazepine exposures for pediatric subjects receiving ESL QD dosing regimens (100–1600 mg, in 100 mg increments) as monotherapy or adjunctive therapy (i.e., with or without concomitant levetiracetam and/or phenobarbital-like AEDs, or carbamazepine). An additional scenario determined typical eslicarbazepine C_min,ss_ in pediatric subjects receiving specific ESL doses in the absence of any concomitant AEDs (Fig. 4). These exposures were matched to adult target exposures (Fig. 4) to propose a pediatric dosing regimen (Table 4). For example, Fig. 4 shows that a typical subject weighing 20 kg would require 400–600 mg ESL QD to achieve eslicarbazepine exposures equivalent to those observed in adults taking ESL 800–1200 mg QD. A sufficient number of subjects were exposed to ESL in Studies BIA-2093-208 and 305 to provide adequate safety data to allow for ESL use in pediatric subjects ≥ 11 kg at dose regimens up to 1200 mg QD.

**Table 4:** Proposed ESL (adjunctive therapy or monotherapy) titration and maintenance dosing regimens for pediatric patients between the ages of 4 and 17 years

Body weight range (kg)	Titration dose (mg/day)	Minimum–maximum maintenance dose (mg/day)^a^
11–21	200	400–600
22–31	300	500–800
32–38	300	600–900
> 38	400	800–1200

Doses were selected to target exposures that are known to be safe and effective in adults

^a^Due to the absence of safety data in pediatric subjects for daily doses above 1200 mg, the maximum proposed maintenance dose in pediatric subjects is 1200 mg QD

*ESL* eslicarbazepine acetate, *QD* once daily

Simulation scenarios for monotherapy or adjunctive therapy with carbamazepine demonstrated that administration of ESL to pediatric subjects, within the selected dose ranges (Table 1), would yield similar eslicarbazepine exposures whether taken as adjunctive therapy or as a monotherapy, and that eslicarbazepine exposures would be comparable to those observed in the adult population with effective and well-tolerated doses of ESL (800–1600 mg QD).

## Discussion

A recent FDA analysis provides evidence across AEDs that exposure–response relationships are preserved between adult and pediatric subjects with POS (4 years of age and older) [2]. We used the results from previous pediatric clinical trials of ESL and the analyses outlined in this manuscript to satisfy the three FDA criteria needed to obtain an indication for ESL in the treatment of POS in pediatric subjects (≥ 4 years). We illustrate the application of adult exposure matching for pediatric dose selection, which was submitted to the FDA as part of the application for a pediatric indication for ESL (adjunctive therapy and monotherapy) in patients 4 years and older with POS, without the need to conduct a controlled clinical trial for efficacy. Furthermore, as previous studies have demonstrated that ESL is well-tolerated in pediatric subjects aged 4–17 years [9–11], we used a PPK modeling and simulation strategy to provide a quantitative basis to support ESL dose selection for pediatric subjects aged 4–17 years.

The final structural PK model was adequately characterized by a one-compartment model with formulation-specific first-order absorption and first-order elimination. In the final model, the typical first-order absorption rate constant (KA) estimates for the tablet and oral suspension formulations were fixed to estimates obtained from the Study 202 data, as there were large differences in KA estimates between studies when these parameters were estimated separately. The KA estimates from Study 202 were from richly sampled data, and considering the lack of formulation differences between studies and the similarity of covariate distributions between studies, were deemed more reliable than the KA estimates obtained from the sparse data collected in Study 305. Body weight was identified as the only statistically significant demographic covariate for clearance and volume of distribution. Allometric scaling with exponents of 0.75 and 1 were used to account for weight-based changes in clearance and volume of distribution, respectively. An alternative model with estimated exponents was evaluated, but not selected (as per recommendations in Holford et al. [22], and Anderson and Holford [23]) due to the potential for imprecision of empirical estimates from typical sized datasets with limited numbers of subjects, and the proximity of the estimate exponents to theoretical values (e.g., 0.648 vs 0.75 for the power on CL, and 1.19 vs 1 for the power on V).

The covariate analysis concluded that concomitant use of carbamazepine, levetiracetam, and phenobarbital-like AEDs with ESL are significant determinants of eslicarbazepine PK in pediatric subjects. The model estimated that concomitant use of carbamazepine with ESL would decrease the bioavailability, and thus the overall exposure, of eslicarbazepine. Concomitant use of phenobarbital-like AEDs with ESL would increase the clearance of eslicarbazepine, and would therefore also decrease the overall exposure of eslicarbazepine. The predicted effects of carbamazepine and phenobarbital-like AEDs on eslicarbazepine PK were similar for pediatric and adult subjects [12, 13]. Levetiracetam decreased eslicarbazepine clearance in pediatric subjects, but had no statistically significant effect on clearance in adults. However, the small effect of levetiracetam on clearance of eslicarbazepine in pediatric subjects may be an artifact of limited sample size, study design, and/or variability, and may not represent a true difference in clearance. Overall, the parameters of the final PK model were precisely estimated. The final PK model described the observed data well and it was deemed appropriate for use in simulations.

Previously developed adult PPK models [12, 13] were used in model-based simulations to identify adult eslicarbazepine exposures that corresponded with effective and well-tolerated doses of ESL in adults. Additional simulations, using the pediatric PPK model, were used to determine typical eslicarbazepine concentrations in pediatric subjects aged 2–17 years receiving specific ESL doses. Targeted exposure matching (matching of pediatric eslicarbazepine exposures to eslicarbazepine exposures in adults taking effective and well-tolerated doses of ESL) was then used to determine an appropriate dosing regimen for pediatric subjects aged 4–17 years (Table 1). Among the different measures of eslicarbazepine exposure in adults, the relationship with efficacy was strongest for C_min,ss_, which was therefore used as the metric for targeted exposure matching in the pediatric PPK model. Previously it was reported that C_max_ is not a statistically significant predictor of common adverse events associated with ESL use in adults [19]. Nevertheless, C_max_ at the highest approved adult ESL maintenance dose (1600 mg) was considered in setting the upper limit of the targeted exposure range. The proposed doses for subjects aged 4–17 years apply to those taking ESL as an intact tablet, as a crushed tablet, or as an oral suspension, as previous clinical investigations have demonstrated the bioequivalence of these formulations [24–26].

## Conclusions

Overall, the analyses described herein represent a comprehensive effort to use modeling and simulation-based strategies to extrapolate efficacy data from adults to pediatric patients (aged 4–17 years). Furthermore, this analysis supports ESL (adjunctive therapy or monotherapy) dose selection in pediatric patients, without having to conduct an additional US-based clinical trial for efficacy in these patients.

## References

[1] Nunes T, Rocha JF, Falcao A, Almeida L, Soares-da-Silva P (2013). Steady-state plasma and cerebrospinal fluid pharmacokinetics and tolerability of eslicarbazepine acetate and oxcarbazepine in healthy volunteers. Epilepsia.

[2] AAP News (2016) Food and Drug Administration update, 6 April 2016. http://www.aappublications.org/news/2016/04/06/FDAUpdate040616. Accessed 25 Aug 2017

[3] Mehrotra S (2016) Quantitative analysis to support full extrapolation of efficacy in children for partial onset seizures in adjunctive setting: FDA-PEACE initiative, January 2016. http://pharmacy.umaryland.edu/media/SOP/wwwpharmacyumarylandedu/centers/cersievents/pedsextrapolation/mehrotra-presentation-notes.pdf. Accessed 5 May 2017

[4] Men AY (2015) Assessing quality and quantity of data to establish exposure-response similarity between adults and pediatric patients: PEACE initiative, January 2015. https://www.pharmacy.umaryland.edu/media/SOP/wwwpharmacyumarylandedu/centers/cersievents/pediatricpbpk/Men%20-%20PEACE%20Initiative.pdf. Accessed 5 May 2017

[5] Pellock JM, Carman WJ, Thyagarajan V, Daniels T, Morris DL, D’Cruz O (2012). Efficacy of antiepileptic drugs in adults predicts efficacy in children: a systematic review. Neurology.

[6] HHS, FDA, CDER, and CBER (2003) Guidance for industry. Exposure-response relationships—study design, data analysis, and regulatory applications. https://www.fda.gov/downloads/drugs/guidancecomplianceregulatoryinformation/guidances/ucm072109.pdf. Accessed 29 Jan 2018

[7] Dunne J, Rodriguez WJ, Murphy MD, Beasley BN, Burckart GJ, Filie JD, Lewis LL, Sachs HC, Sheridan PH, Starke P, Yao LP (2011). Extrapolation of adult data and other data in pediatric drug-development programs. Pediatrics.

[8] Mulugeta Y, Barrett JS, Nelson R, Eshete AT, Mushtaq A, Yao L, Glasgow N, Mulberg AE, Gonzalez D, Green D, Florian J, Krudys K, Seo S, Kim I, Chilukuri D, Burckart GJ (2016). Exposure matching for extrapolation of efficacy in pediatric drug development. J Clin Pharmacol.

[9] Moreira J, Pinto R, Rocha J, Soares-da-Silva P (2015) Effect of eslicarbazepine acetate on cognition in children with epilepsy. Presented at the World Congress of Neurology, Santiago, Chile, October 31–November 5, 2015

[10] Rocha J, Moreira P, Pinto R, Soares-da-Silva P (2015) A placebo-controlled trial of eslicarbazepine acetate add-on therapy for partial seizures in children. Presented at the World Congress of Neurology, Santiago, Chile, October 31–November 5, 2015

[11] BIAL—Portela & Ca SA, Data on file, S. Mamede do Coronado, Portugal

[12] Lu Q, Ludwig E, Fiedler-Kelly J, Maier G, Blum D, Kharidia J (2013) Population pharmacokinetic evaluation of eslicarbazepine acetate for adjunctive therapy in refractory partial onset seizures. Presented at the AAPS Annual Meeting and Exposition, San Antonio, TX, 10–14 November 2013

[13] Abou-Khalil B, Ali I, Shah A, Fiedler-Kelly J, Ludwig E, Sunkaraneni S, Blum D (2014). Eslicarbazepine acetate monotherapy: a population pharmacokinetic analysis. Epilepsy Curr.

[14] Department of Health and Human Services Food and Drug Administration (2016) Best Pharmaceuticals for Children Act and Pediatric Research Equity Act. July 2016 status report to Congress. https://www.fda.gov/ScienceResearch/SpecialTopics/PediatricTherapeuticsResearch/ucm509707.htm. Accessed 2 Aug 2017

[15] Gidal B, Jacobson M, Ben-Menachem E, Carreño M, Blum D, Soares-da-Silva P, Falcao A, Rocha F, Moreira J, Grinnell T, Ludwig E, Fiedler-Kelly J, Passarell J, Sunkaraneni S (2018). Exposure-safety and efficacy response relationships and population pharmacokinetics of eslicarbazepine acetate. Acta Neurol Scand.

[16] Schwartz GJ, Brion LP, Spitzer A (1987). The use of plasma creatinine concentration for estimating glomerular filtration rate in infants, children, and adolescents. Pediatr Clin N Am.

[17] Bergstrand M, Hooker AC, Wallin JE, Karlsson MO (2011). Prediction-corrected visual predictive checks for diagnosing nonlinear mixed-effects models. AAPS J.

[18] Rogin J, Cole A, Strom L, Passarell J, Fiedler-Kelly J, Ludwig E, Blum D, Sunkaraneni S (2015). Relationship between exposure and efficacy of eslicarbazepine acetate monotherapy. Epilepsy Curr.

[19] Sunkaraneni S, Ludwig EA, Passarell JA, Blum D, Grinnell T, Fiedler-Kelly J (2018). Population pharmacokinetics and exposure-response analyses of eslicarbazepine acetate efficacy and safety in monotherapy of partial-onset seizures. J Clin Pharmacol.

[20] Gibaldi M, Perrier D (1982). Pharmacokinetics.

[21] Centers for Disease Control and Prevention (CDC) (2009) Percentile data files with LMS values [data files on the Internet] Atlanta (GA) [updated 4 Aug 2009]. https://www.cdc.gov/growthcharts/percentile_data_files.htm. Accessed 25 Aug 2017

[22] Holford N, Heo YA, Anderson B (2013). A pharmacokinetic standard for babies and adults. J Pharm Sci.

[23] Anderson BJ, Holford NH (2008). Mechanism-based concepts of size and maturity in pharmacokinetics. Annu Rev Pharmacol Toxicol.

[24] Falcao A, Lima R, Sousa R, Nunes T, Soares-da-Silva P (2013). Bioequivalence of eslicarbazepine acetate from two different sources of its active product ingredient in healthy subjects. Drugs R&D.

[25] Fontes-Ribeiro C, Nunes T, Falcao A, Neta C, Lima R, Tavares S, Almeida L, Macedo T, Soares-da-Silva P (2005). Eslicarbazepine acetate (BIA 2-093): relative bioavailability and bioequivalence of 50 mg/mL oral suspension and 200 mg and 800 mg tablet formulations. Drugs R&D.

[26] Sunkaraneni S, Kharidia J, Schutz R, Blum D, Cheng H (2016). A pharmacokinetic study comparing eslicarbazepine acetate administered orally as a crushed or intact tablet in healthy volunteers. Clin Pharmacol Drug Dev.

